# Effects of Piperazine Derivative on Paclitaxel Pharmacokinetics

**DOI:** 10.3390/pharmaceutics11010023

**Published:** 2019-01-08

**Authors:** Jaeok Lee, Song Wha Chae, A Reum Oh, Ji Hye Yoo, Hea-Young Park Choo, Sandy Jeong Rhie, Hwa Jeong Lee

**Affiliations:** 1 College of Pharmacy and Graduate School of Pharmaceutical Sciences, Ewha Womans University, Seoul 03760, Korea; leejo@ewha.ac.kr (J.L.); yuki1226@naver.com (S.W.C.); dhdkf9922@ewhain.net (A.R.O.); jibong9077@naver.com (J.H.Y.); hypark@ewha.ac.kr (H.-Y.P.C.); 2 College of Pharmacy and Division of Life and Pharmaceutical Sciences, Ewha Womans University, Seoul 03760, Korea; jrhiepa@gmail.com

**Keywords:** piperazine derivatives, P-glycoprotein inhibitor, pharmacokinetics, bioavailability, paclitaxel

## Abstract

Paclitaxel (PTX) is an anticancer agent that is used to treat many cancers but it has a very low oral bioavailability due, at least in part, to the drug efflux transporter, P-glycoprotein (P-gp). Therefore, this study was performed to enhance oral bioavailability of PTX. In this study, we investigated the effects of several piperazine derivatives on P-gp function in vitro. Compound **4** was selected as the most potent P-gp inhibitor from the in vitro results for examining the pharmacokinetic (PK) changes of PTX in rats. Compound 4 increased the AUC_inf_ of PTX without alterations in the *C*_max_ value. The elimination half-life was extended and the oral clearance decreased. Additionally, the *T*_max_ was delayed or widened in the treatment groups. Therefore, the bioavailability (BA) of PTX was improved 2.1-fold following the co-administration of 5 mg/kg of the derivative. A piperazine derivative, compound 4, which was confirmed as a substantial P-gp inhibitor in vitro increased the BA of PTX up to 2-fold by a lingering absorption, in part due to inhibition of intestinal P-gp and a low oral clearance of PTX. These results suggest that co-administering compound **4** may change the PK profile of PTX by inhibiting P-gp activity in the body.

## 1. Introduction

Paclitaxel (PTX) is a widely-used anticancer drug that is known to have poor bioavailability (below 10%) due to its low solubility and membrane permeability [1]. Further, the interaction of PTX with the membrane drug efflux transporter functions as an additional barrier to gastrointestinal (GI) PTX absorption [2]. It has also been reported that approximately 50% of orally administered PTX is excluded from the GI track by P-glycoprotein (P-gp) [3]. There have been many studies on how to improve the bioavailability (BA) of PTX by inhibiting P-gp function and expression over the last two decades [2,4,5,6,7,8,9].

P-gp, a well-known crucial member of the ATP-binding cassette (ABC) superfamily and a well-characterized transporter, has broad substrate specificity and affects the pharmacokinetic (PK) behavior of many drugs that are P-gp substrates. It is highly expressed in important organs such as the intestine [10], liver [11], and kidney [12], which are involved in drug absorption, metabolism and excretion. Because the efflux transporter is the primary cause of multi-drug resistance (MDR) and alters the pharmacokinetics of various drugs, P-gp modulation could counter the MDR and increase the BA of orally delivered drugs. Identification and development of novel P-gp modulators have therefore been studied for over three decades [13,14].

Piperazine is an important six-membered, N-heterocyclic compound found in the structure of a number of pharmaceuticals used against many different diseases, such as multiple cancers, primary hypertension, HIV/AIDS, and inflammation [15,16]. However, only a limited number of studies have assessed P-gp modulation with piperazine or its derivatives. A five-hydroxychromone linked piperazine unit was identified as a highly active P-gp-mediated MDR modulator [17]. Some novel compounds with 10-beta-phenyl ethers of dihydroartemisinin (DHA) with piperazine substitutions have demonstrated significant effects on transporter-mediated antiproliferation in human breast cancer cells. Among them, two DHA compounds with piperazine substitution had a 1.5–2-fold greater effect on antiproliferative activity than the DHA compounds without piperazine substitution [18]. These reports have suggested the possibility of piperazine or its analogues as potential P-gp modulators.

**Figure 1:**
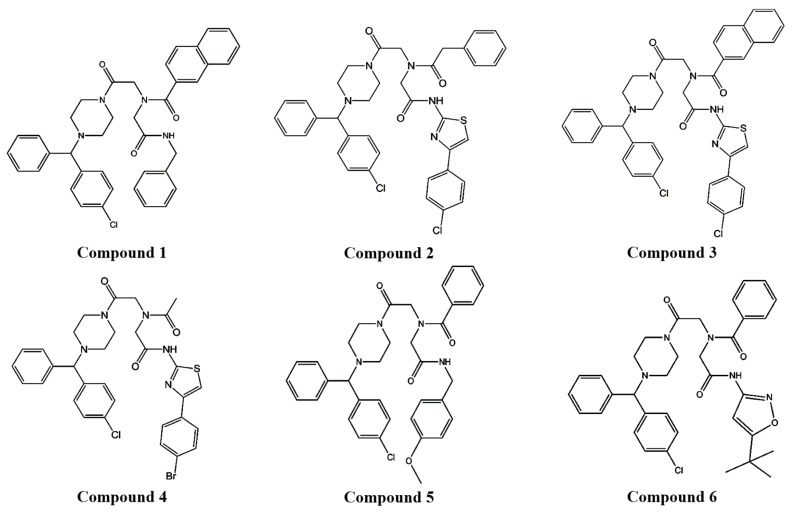
The structures of piperazine derivatives.

We demonstrated that compound 4, piperazine iminodiacetic acid triamide, which was selected as a potential P-gp inhibitor from several piperazine derivative candidates in vitro (Figure 1), increased the BA of PTX, known to have poor BA (below 10%), as well as a P-gp substrate anticancer drug [1] with a draggy absorption and a slow elimination in rats.

## 2. Materials and Methods

### 2.1. Materials

Synthesized piperazine derivatives were provided by Dr. Hea-Young Park Choo [19]. Daunomycin (DNM) and [^3^H]-DNM (16 Ci/mmol) were purchased from Merck (Darmstadt, Germany) and PerkinElmer Life and Analytical Sciences (Boston, MA, USA), respectively. PTX was obtained by Samyang Genex (Daejeon, Korea), and (±)-Verapamil hydrochloride was provided by Sigma-Aldrich (St. Louis, MO, USA). Human P-gp membrane and BD Gentest^TM^ ATPase assay kits were purchased from BD Bioscience (Woburn, MA, USA). All reagents and solvents were commercially provided at molecular biology and cell culture grades or HPLC analysis grade. A P-gp overexpressing cell line, MCF-7/ADR, was provided by Dr. Marilyn E. Morris (University of Buffalo, NY, USA). Laboratory animals were purchased from Orient Bio (Seongnam, Korea).

### 2.2. Cell Culture

The MDR human breast cancer cell line MCF-7/ADR was maintained in RPMI 1640, supplemented with 2 mM L-glutamine, 10 mM HEPES, 24 mM NaHCO_3_, 10% fetal bovine serum, and 1% antibiotic-antimycotic agent at 37 °C in a humidified 5% CO_2_ atmosphere.

### 2.3. Cytotoxicity Assay

The cytotoxicity of each piperazine derivative was examined in the cells using the sulforhodamine B (SRB) assay [20]. The changes in IC_50_ values of DNM, P-gp substrate anticancer drug were estimated in the presence of piperazine derivatives at a concentration of 100 μM. The details of the cytotoxicity assay were described in our previous report [9,21]. Verapamil (VER), a representative P-gp substrate and inhibitor, was used as a positive control at a concentration of 100 μM. The IC_50_ values were calculated with the Table Curve Windows program. The assay was performed in triplicate.

### 2.4. [^3^H]-DNM Uptake and Efflux Studies

Tritium-labeled P-gp substrate uptake and efflux studies were performed using the modified Harker and Sikic method [22]. The pre-selected derivatives, compounds 2 and 4, were applied at a final concentration of 100 μM with 0.05 μM of tritium-labeled DNM for 2 h for the uptake study. For the efflux study, cells were incubated with 0.05 μM tritium-labeled DNM for 1 h and subsequently in the absence or presence of 100 μM of pre-selected derivatives for another 1 h after removing 0.05 μM of the tritium-labeled DNM. The uptake and efflux values based on the radioactivity estimated by Liquid Scintillation Counting were normalized to those of 1 × 10^6^ cells. The detailed procedures have been reported previously [9,21,23]. All the experiments were performed in triplicate and verapamil (VER) was used as a positive control.

### 2.5. P-Glycoprotein ATPase Activity Assay

Using human P-gp membrane, the P-gp ATPase activity of pre-selected derivatives, compounds 2 and 4, was estimated at a concentration of 100 μM [9,21,23]. The rate of phosphate release per mg membrane protein represented the ATPase activity by converting to the relative ratio versus control value. The assay was performed in duplicate and VER, a P-gp inhibitor and ATPase stimulator was used as the positive control.

### 2.6. Animals

All the animal experiments were performed using six-week-old male Sprague-Dawley rats (200–235 g) under the procedures granted by the Ewha Womans University Institutional Animal Care and Use Committee (IACUC) (No. 2012-01-019), Korea [23].

### 2.7. Pharmacokinetic Studies

PTX at 2 mg/mL was prepared immediately prior to use with a commercial formulation of Taxol^®^ (Cremophor^®^ EL, anhydrous ethanol and isotonic saline (1:1:4, *v*/*v*/*v*)) [23]. Compound 4 (*N*-(4-(4-bromophenyl)thiazol-2-yl)-2-(*N*-(2-(4-((4-chlorophenyl)(phenyl)methyl)piperazin-1-yl)-2-oxoethyl)acetamido)acetamide) was prepared using a process similar to PTX formulation for oral administration.

For intravenous injection of 2 mg/kg of PTX (IV) and oral administration of 25 mg/kg of PTX (PO) with 0, 2, or 5 mg/kg of compound 4, the rats were divided into 4 groups on the day. The common carotid artery was catheterized for blood sampling. Blood sampling was conducted at 0, 0.033, 0.083, 0.25, 0.5, 1, 2, 3, 4, 6, 10, and 24 h for the IV group and 0, 0.25, 0.5, 1, 2, 3, 4, 6, 8, 10, and 24 h for PO groups [21,23].

Prepared plasma samples mixed with internal standard (4-hydroxybenzoic acid n-hexyl ester) were analyzed by the HPLC system [21,23].

WinNonlin^®^ Professional version 5.2 software (Pharsight Corporation, Mountain View, CA, USA) was used to estimate the PK parameters following intravenous or oral PTX administrations to the rats. Non-compartmental analysis was performed using the plasma PTX concentration–time profiles to obtain the following PK parameters: Area under the plasma concentration–time curve from zero time to infinity (AUC_inf_), elimination half-life (*t*_1/2_), apparent volume of distribution after oral administration (Vz/F), and oral clearance (Cl/F). The maximum plasma concentration (*C*_max_) and the time required to reach *C*_max_ (*T*_max_) were directly measured from the plasma PTX concentration–time curve. The absolute BA and relative BA (AB and RB, respectively) of PTX were calculated using the respective formulas below:

AB (%) = AUC po/AUC iv × IV dose/PO dose × 100

RB (%) = AUC po co–administration/AUC po control × 100

### 2.8. Statistical Analysis

Tukey’s HSD test in conjunction with an ANOVA was used for statistical analysis of uptake and efflux and for pharmacokinetics studies. Statistical significance was represented as ‘*p* value < 0.05.’

## 3. Results

### 3.1. Effect of Piperazine Derivatives on Uptake and Efflux of P-gp Substrate

To determine the influence of the derivatives on P-gp function, a P-gp substrate drug, DNM was applied with piperazine derivatives to MCF-7/ADR cells. Initially, four of 6 piperazine derivatives (Figure 1) were selected to examine their effect on DNM, a P-gp substrate drug, cytotoxicity in P-gp-overexpressing cells, because the selected derivatives, compounds 1–4 were not toxic to the cells at five different concentrations (5, 10, 25, 50, and 100 μM), but compounds 5–6 were toxic to the cells to some degree (Table S1).

All the tested derivatives decreased the IC_50_ value of DNM to less than half that with the negative control (31.7 μM) (Table 1). Compounds 1, 2, and 4 reduced the DNM IC_50_ values (5.7, 7.6 and 2.7 μM, respectively) similarly to the positive control, VER (3.1 μM). Among all derivatives, compound 4 appeared to be the most effective for reducing the IC_50_ value of the P-gp substrate drug. Although compound 1 decreased the IC_50_ value of DNM more than compound 2, compound 2 was relatively less toxic to the cells at all concentrations tested, from 5 μM to 100 μM (Table S1). Based on these results, compounds 2 and 4, which had similar structures, were selected for further in vitro studies.

**Figure 2:**
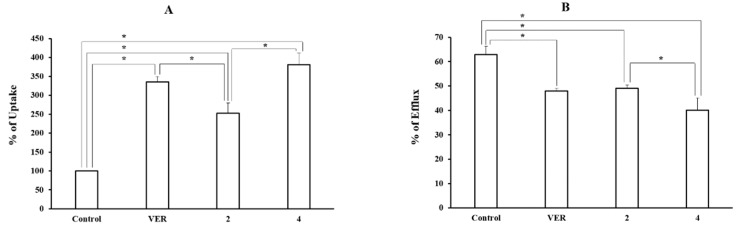
The Effect of 2 pre-selected derivatives on the uptake (**A**) and efflux (**B**) of [^3^H]-DNM in MCF-7/ADR cells. [^3^H]-DNM uptake and efflux were assayed after the incubation with each derivative (100 μM) for 2 h and 1 h, respectively. VER was used as a positive control. Control represents uptake or efflux of tritium-labeled P-gp substrate alone. Each data represents the mean ± S.D. (*, *p* < 0.05) from triplicate experiments.

The pre-selected two piperazine derivatives (compounds 2 and 4) were used to measure the cellular uptake and efflux of tritium-labeled DNM at a concentration of 100 μM in order to confirm the P-gp inhibitory function (Figure 2). Both derivatives significantly increased the tritium-labeled DNM uptake to 2.5–3.8-fold (252.9 ± 26.8% and 380.8 ± 31.5% for compounds 2 and 4, respectively) compared with the control and decreased the efflux up to 0.6–0.8 fold (40.2 ± 4.9% and 49.1 ± 1.4% for compounds 4 and 2, respectively) compared with the control (63.0 ± 3.3%). Compound 4 showed a more significant effect on DNM uptake and efflux than compound 2. Compound 4 showed a stronger effect on both uptake and efflux functions of the P-gp substrate compared to VER, the positive control (335.1 ± 14.7% in uptake and 48.0 ± 1.1% in efflux).

### 3.2. Effect of Piperazine Derivatives on P-gp ATPase Activity

To examine the modulation mechanism on P-gp activity, the effect of compounds 2 and 4 on ATPase activity was estimated using an ATPase assay kit and a human P-gp membrane (Table 2). Compounds 2 and 4 did not exhibit a significant modulating effect on ATPase activity (1.3- and 1.2-fold, respectively) compared to the control, whereas VER (100 µM) increased the activity by more than 2-fold compared to the control.

### 3.3. Effect of Compound 4 on P-gp Function In Vivo: PTX Pharmacokinetics

**Figure 3:**
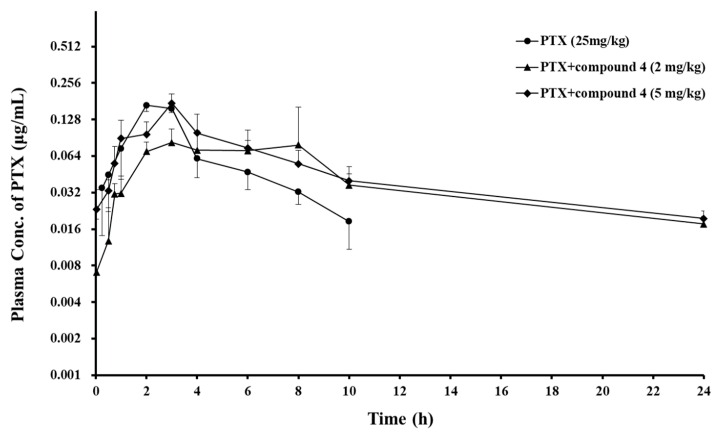
Mean plasma concentration–time profiles of the oral co-administration of PTX with piperazine derivative, compound 4 to rats. Bars represent S.D. (*n* = 3–4). (●) PTX (25 mg/kg, PO) alone, PTX (25 mg/kg) co-administrated with compound **4** (▲) 2, or (◆) 5 mg/kg.

Based on the results from the in vitro studies, compound 4 was selected to examine the P-gp modulating effect in an animal model. PTX was orally administered to rats to determine the effect of compound 4 on the PK profile of the P-gp substrate drug. Figure 3 and Table 3 show the mean plasma PTX concentration–time profiles and PK parameters of PTX, respectively. The AUC_inf_ and t_1/2_ of the anticancer drug increased with an increasing dose of compound 4, whereas the total clearance was reduced with an increasing dose of the compound. The *C*_max_ and Vz/F following oral administration of PTX were not significantly different among the control group and treatment groups, but *T*_max_ was delayed or widened. As a result, the BA of PTX was enhanced 1.6- and 2.1-fold at 2 and 5 mg/kg doses of compound 4, respectively (Table 3).

## 4. Discussion

We selected the synthesized piperazine derivative, *N*-(4-(4-bromophenyl)thiazol-2-yl)-2-(*N*-(2-(4-((4-chlorophenyl)(phenyl)methyl)piperazin-1-yl)-2-oxoethyl)acetamido)acetamide (compound 4) as a potential P-gp inhibitor based on in vitro results. The effect of compound 4 on the BA of PTX was then monitored. Compound 4 increased the BA of the anticancer drug PTX by as much as 56–106.6%, where the greatest effect was seen when the compound was co-administered orally at a dose of 5 mg/kg (Table 3). The absolute BA of PTX was only 7.1% when it was orally administered alone.

P-gp modulation mediated by piperazine derivatives has not been studied substantially, and most studies have been performed in vitro. Therefore, P-gp inhibition by piperazine or its derivatives has not yet been elucidated. One study reported that a radio-iodinated piperazine derivative, 5-[3-{4-(2-phenyl-2-(4-[125I]iodo-phenyl)acetyl)piperazin-1-yl}-2-hydroxypropoxy] quinoline, induced approximately a 10-fold shorter absorption half-life and a 50% shorter mean residence time in xenograft mice inoculated with P-gp overexpressed in human uterine sarcoma cells (MES-SA/Dx5) than those in the parental cells [24]. This observation was differed from our results, which could be attributed to different animal species, chemical structure, and/or formulation.

However, although piperazine or its derivatives did not demonstrate a powerful effect on P-gp modulation, it is still considered a potential approach because piperazine is the basic core structure of a number of pharmaceuticals for many diseases [15,16]. Moreover, in vitro investigations have shown the possibility of piperazine or derivative use for modulating MDR mediated by P-gp [17,18] and other ABC transporters, such as breast cancer resistance protein (BCRP) [25] and multidrug resistance-associated protein 1 (MRP1) [26].

In the present study, compounds 1–4 (Figure 1) were selected to examine their effect on DNM cytotoxicity in MCF-7/ADR cells. These selected compounds were not toxic to the P-gp overexpressing cells at five different concentrations (5, 10, 25, 50, and 100 μM) (Table S1). Previously, we examined P-gp inhibitory effect of a positive control, VER at two different concentrations (50 and 100 uM) in in vitro studies. Because VER at the concentration of 50 uM did not fully inhibit P-gp function, as compared to 100 uM concentration, based on the results of cytotoxicity and uptake studies [9,27], the cells were treated with 100 uM of the piperazine derivatives and VER to investigate their P-gp inhibitory activity in this study. Among the four piperazine derivatives, the chemical structures of compounds 2–4 were similar (Figure 1). Based on chemical structure and reversing effect on resistance to DNM by decreasing IC_50_ value in the cells, compounds 2 and 4 were chosen to examine their effects on uptake and efflux functions of P-gp substrate and P-gp ATPase activity.

The PK parameters of PTX provided more information about the P-gp modulating effect of the piperazine derivative. Although the *C*_max_ and Vz/F of PTX were not considerably altered after oral co-administration with compound 4, the increased AUC_inf_, prolonged *T*_max_ and *t*_1/2_, and decreased oral clearance of PTX (Table 3) in the presence of compound 4 may result in an enhanced BA of PTX by inhibiting P-gp located in the absorption and elimination organs.

The mean plasma concentration–time profiles of PTX provided additional information and the following points were observed (Figure 3). In the absorption phase of PTX, the piperazine derivative induced to extend or drag the PTX absorption peak in the treatment groups, and in the PTX elimination phase, the derivative led to slower elimination of PTX. Because P-gp is located in whole intestine [28], inhibition of P-gp function by the piperazine derivative can occur through whole intestine, which can induce a lingering absorption peak. This kind of peak was also previously observed when PTX was orally administered with VER at a dose of 5 mg/kg [29].

Compound 4 could affect not only the PTX absorption pathway but also the elimination pathway. The P-gp efflux transporter is related to drug elimination as well as drug absorption following oral administration [2,30]. PTX is eliminated by the hepatic metabolism, renal excretion [30,31], and biliary excretion [32]. Because P-gp is expressed in the hepatic canalicular membrane [11] and the proximal tubule of the kidney [12], the interaction between PTX and compound 4 in the P-gp efflux function can occur, resulting in slow elimination of PTX by P-gp inhibition in the eliminating organs.

## 5. Conclusions

Four piperazine derivatives showed a P-gp inhibitory activity in cell studies. Among them, compound 4, *N*-(4-(4-bromophenyl)thiazol-2-yl)-2-(*N*-(2-(4-((4-chlorophenyl)(phenyl)methyl)piperazin-1-yl)-2-oxoethyl)acetamido)acetamide, was selected as the most potent P-gp inhibitor candidate from in vitro studies. The results indicated that co-administration of 5 mg/kg of compound 4 significantly enhanced the oral BA of PTX by 2.1-fold in rats. Although the interaction between PTX and compound 4 was not fully understood in vivo, this result may be, at least in part, possibly due to the inhibition of P-gp present in absorption and elimination organs. Therefore, the piperazine derivative can be exploited in order to enhance the oral BA of other P-gp substrate drugs as well as PTX.

## Figures and Tables

**Table 1:** The change in DNM IC_50_ value by 100 μM piperazine derivatives in vitro (The numerical data were represented the cell survival ratio).

Category	Derivatives	DNM IC_50_ (μM)
Control	Negative control	31.7
	Positive control *	3.1
Piperazine derivatives	1	5.4
	2	7.6
	3	11.5
	4	2.7

* Positive control: VER, a P-gp inhibitor.

**Table 2:** Effect of piperazine derivatives and verapamil on P-gp ATPase activity.

Comp Conc. (μM)	Blank	VER ^a^	2	4
100	1.00	2.24	1.29	1.18

^a^ VER: ATPase stimulator; the values are expressed as a ratio of the control (blank).

**Table 3:** PK parameters of PTX orally co-administrated with compound 4 (2 and 5 mg/kg, PO) in rats.

PK Parameters	PTX Oral Control	Co-Administration of PTX with Compound 4		PTX IV Control
		2 mg/kg	5 mg/kg	
Cmax (ng/mL)	189 ± 76	112 ± 55	175 ± 0.032	2968 ± 428
Tmax (h)	2.0	[2.0–8.0]	3.0	
AUCinf (ng·h/mL)	745 ± 208	1162 ± 322	1539 ± 209 *	835 ± 155
*t*_1/2_ (h)	3.1 ± 0.8	7.9 ± 2.0 *	11.0 ± 2.7 *	2.06 ± 0.8
ke (1/h)	0.233 ± 0.062	0.092 ± 0.027 *	0.066 ± 0.016 *	0.37 ± 0.12
Vz/F (mL)	40,589 ± 16,353	56,582 ± 25,172	53,466 ± 15,335	1754 ± 419
Cl/F (mL/h)	8721 ± 2040	4811 ± 1406 *	3356 ± 455 *	614 ± 123
AB (%)	7.1	11.1	14.7	100.0
RB (%)		156.0	206.6 *	

(* *p* < 0.05 compared with PTX oral control). Data are presented as mean ± S.D. (3–4 rats per group).

## Supplementary Materials

The following are available online at http://www.mdpi.com/1999-4923/11/1/23/s1, Table S1. The toxic effect of each derivative in MCF-7/ADR cells after 2 h incubation.
