# Supplementary material for: Effects of Piperazine Derivative on Paclitaxel Pharmacokinetics
Source: Pharmaceutics. 2019 Jan 8;11(1):23. doi: 10.3390/pharmaceutics11010023 (PMC6359037; doi:10.3390/pharmaceutics11010023)
Supplement: Supplementary file 1 [file pharmaceutics-11-00023-s001.pdf]

pharmaceutics

Supplementary Materials: Effects of piperazine derivative on paclitaxel pharmacokinetics

Jaeok Lee, Song Wha Chae, A Reum Oh, Ji Hye Yoo, Hea-Young Park Choo, Sandy Jeong Rhie, and Hwa Jeong Lee

Table S1. The toxic effect of each derivative in MCF-7/ADR cells after 2 h incubation.

Comp Conc. (μM)	1	2	3	4	5	6
**5**	89.77	102.70	102.03	100.76	101.71	100.72
**10**	90.47	102.87	101.69	98.50	87.22	101.38
**25**	89.23	86.94	102.24	96.14	83.46	100.76
**50**	88.56	86.10	86.26	96.90	83.84	85.68
**100**	87.21	87.75	85.90	87.63	81.94	82.91

The numerical data were represented the cell survival ratio.
